# Comparison of Polysaccharides Extracted from Cultivated Mycelium of *Inonotus obliquus* with Polysaccharide Fractions Obtained from Sterile Conk (Chaga) and Birch Heart Rot

**DOI:** 10.3390/jof7030189

**Published:** 2021-03-08

**Authors:** Gabriele Beltrame, Jani Trygg, Jarl Hemming, Zenghua Han, Baoru Yang

**Affiliations:** 1 Food Chemistry and Food Development, Department of Life Technologies, University of Turku, Itäinen Pitkäkatu 4, FI-20520 Turku, Finland; gabbel@utu.fi (G.B.); jani.trygg@utu.fi (J.T.); 2 Wood and Paper Chemistry, Åbo Akademi University, Porthaninkatu 3, FI-20500 Turku, Finland; Jarl.Hemming@abo.fi; 3 Institute of Microbiology, Heilongjiang Academy of Sciences, Zhaolin Street 68, Harbin 150020, China; hanzenghua77@126.cn

**Keywords:** *Inonotus obliquus*, mycelium, birch, polysaccharides, heart rot, β-glucan

## Abstract

The polysaccharides of the sterile conk of *Inonotus obliquus* (Chaga) have demonstrated multiple bioactivities. The mycelium of this basidiomycete, obtained after submerged cultivation, has been considered a feasible alternative to the sterile conk for the production of polysaccharides. However, previous research has paid little attention to the differences in the structures of polymers obtained from the different resources. Moreover, the birch wood colonized by *I. obliquus* has never been investigated as a source of bioactive polysaccharides. In the present study, polysaccharide fractions produced from cultivated mycelium, sterile conks of different geographical origins, and birch heart rot were investigated. High amounts of phenolic compounds, possibly lignans, were bound to the sterile conk polysaccharides. Mycelial polysaccharides were rich in α- and β-glucans and had high (10^5^ Da) and low (10^4^ Da) molecular weight populations. On the other hand, sterile conk polysaccharides were mainly β-glucan of lower and monodispersed molecular weight (10^3^ Da). Heart rot polysaccharides were comprised mainly of low molecular weight (10^3^ Da) hemicelluloses. Nevertheless, fungal polysaccharides were identified in the extracts. The differences in structure and molecular properties among the polysaccharide fractions of mycelium, heart rot, and sterile conk are likely associated with differences in bioactivities and, therefore, in nutraceutical potential.

## 1. Introduction

*Inonotus obliquus* is a basidiomycetes of the family Hymenochetaceae (Hymenochetales) of circumboreal distribution, usually found above the 40th parallel north in United States, Canada, Scandinavia, North-Eastern Europe, Baltic countries, Siberia and Northern China. It is an obligate parasite of the birch tree, classified as a white-rot fungi, producing resupinate basidiomes below the birch bark only once in the life cycle, at the death of the host. Its most common feature is a black cracked-shape protrusion from the bark, resulting from the interaction between the host and the parasite. This sterile conk, commonly called Chaga, is composed of sclerotial hyphae and wood, although its exact nature is still poorly understood [1]. The utilization of Chaga as a traditional folk remedy goes back to the 16th century. Its extracts, particularly decoctions, have been used to treat a multitude of diseases, including gastro-intestinal and liver problems, tubercolosis and other infections, and cancer [2]. Starting from ethnopharmacological evidence, multiple studies have focused on the bioactive components of Chaga, with a great deal of attention given to its polysaccharides [3]. Over the years, the polysaccharides extracted from Chaga have been shown, among the available evidence, to scavenge radicals and exert antioxidant activity, to inhibit tumor growth in vitro and in vivo, to activate macrophages and promote cytokine production, and in general to exert immunomodulative activity [4]. These properties are dependent on polysaccharide properties, such as monomer composition, molecular weight, and type of glycosidic linkages [5].

The interest of nutraceutical and food supplement companies for this forest resource has steadily increased in the recent years. However, the harvesting of wild Chaga from birch tree is not industrially sustainable due to the slow growth of Chaga (1–2 cm/year). Moreover, concerns on the impact of excessive harvesting of the sterile conk on the conservation of *I. obliquus* itself have been recently raised [6]. In addition, there are logistical difficulties related to its collection and transportation. One strategy to overcome such issues is the cultivation of the mycelium of *I. obliquus* in liquid medium (submerged cultivation). The advantages of this technique are shorter cultivation time, lower space requirements, lower costs, and easy supplementation of stimulants to increase the yield of cultivation [7,8]. The polysaccharides extracted from the cultivated mycelium have been shown to possess biological activities [9]. The use of a different starting material would require the characterization of the produced polysaccharides and the comparison with those extractable from the sterile conk. There are multiple reports available in the literature on the cultivation of the mycelium of *I. obliquus* for the production of polysaccharide fractions [10]. However, to the best of our knowledge, the structural characterization of polysaccharides extracted from the mycelia obtained from the submerged cultivation of *I. obliquus* has not been published to date.

Another strategy for the sustainable production of nutraceuticals based on Chaga polysaccharides would be the cultivation of the sterile conk. Such methodology, compared to submerged cultivation, would produce a polysaccharide source more similar to the sterile conk found in the wild. However, the research on this strategy is still in its infancy. Some research groups have reported the growth of sterile conk on birch trees after the inoculation of cultured spawn. The growth rate of the sterile conk resulted to be strain-specific [11]. Moreover, Han and coworkers have reported the cultivation of *I. obliquus* sclerotium in cultivation bags, using a substrate based on birch sawdust [12]. After harvesting of the sterile conks, due to the extensive fungal degradation, the remaining birch would be considered as waste, similarly to the spent substrate of mushroom cultivation. Research has already given some attention to the reuse of the solid cultivation substrate for enzyme or polysaccharide production [13,14], due to the mycelial colonization of the lignocellulose material. However, the potential of the birch heart rot as a source of polysaccharides has never been investigated.

The aim of the present work was to extract and characterize the polysaccharides extractable from the cultivated mycelium of *I. obliquus* in terms of monomer composition, molecular weight, and structure. Their properties were compared with polysaccharides extracted from *I. obliquus* sterile conk. For comparison purposes, Chaga obtained from Finnish and Chinese forests were used as the starting material. Moreover, polysaccharides were extracted and analyzed, with the same methodologies, from the heart rot of birch infected by *I. obliquus*, with the aim of assessing the potential of this side-stream as a source of bioactive polysaccharides.

## 2. Materials and Methods

### 2.1. Starting Material

#### 2.1.1. Chaga and Infected Birch Stem

Milled powder of wild Chaga harvested from the Finnish forests was obtained from Eevia Oy (Kauhajoki, Finland). Birch stem infected with *I. obliquus* was obtained from a private forest in the municipality of Lieto, South-West Finland. The heart rot of the birch stem was carved and ground. A milled sample of wild Chaga from the forest of northern China was obtained from Yichun (Heilongjiang, China) and analyzed separately.

#### 2.1.2. Mycelium Cultivation

The strain of *I. obliquus* was isolated from the sterile conk found in Yichun and used for submerged cultivation in the present work. It was deposited at the Institute of Microbiology of the Heilongjiang Academy of Sciences (Harbin, China). The strain was maintained on potato dextrose agar slants. The aerial hyphae were removed from the slant before cultivation. The mycelium was produced by transferring a section of slant into 200 mL of cultivation medium (g/L glucose 15, maltose 15, peptone 2, beef extract 1.3, MgSO_4_·7 H_2_O 1.5; KH_2_PO_4_ 3; vitamin B_1_ 0.01), which was cultured at 27 °C with a shaking speed of 140 rpm for 200 or 250 h. At the end of cultivation time, mycelium was filtered with a mesh, washed with distilled water, oven dried, and ground.

In parallel, mycelium cultivation medium was supplemented with sea buckthorn press cake, aiming to increase the cultivation yield, at different dosages. [15]. After the measurement of the yield of mycelia, the mycelia obtained without supplement and with supplementation dosage of 2.5 g/L were pooled and used for polysaccharide extraction. The pool was labeled as “sb0–2.5”.

### 2.2. Polysaccharide Extraction and Purification

Polysaccharides were extracted from milled wild Finnish Chaga, ground birch heart rot and ground *I. obliquus* mycelium using the same protocol. To remove the lipids and phenolic compounds, the starting material was first extracted three times with 95% technical ethanol at room temperature for 6 h, using a solid/solvent (*w*/*v*) ratio of 1:5. The residue was then extracted three times with deionized water at 100 °C using the same solid/liquid ratio and extraction time as described for ethanol extraction. The extracts of hot water extractions were combined, filtered, and eventually centrifuged to remove the solid material, yielding the hot water extracts. The extraction residue was further subjected to alkali extraction using 2% KOH aqueous solution at 80 °C for 3 h three times, with a solid/solvent (*w*/*v*) ratio of 1:5. Extracts were combined, filtered, and eventually centrifuged to remove the solid material. The alkali extracts obtained were neutralized with acetic acid before purification. Polysaccharides were extracted from milled wild Chinese Chaga with double distilled water (1:40 *w*/*v*) at 80 °C for 1.5 h. Extraction was performed twice, and the extracts were combined. The polysaccharides were precipitated from water and alkali extracts with the addition of three volumes of 95% technical ethanol. Precipitates were recovered after overnight storage at 4 °C with ultracentrifugation. After dissolution in the minimum amount of deionized water, proteins were removed by mixing the solutions multiple times in a separatory funnel with the Sevag mixture (choloroform:n-butanol 5:1) and discarding lower and middle layers [16]. Extracts were then dialyzed (cutoff 12–14 kDa) for 24 h with deionized water to remove small molecules, such as free phenolics, sugars, and small peptides. The solutions were then subjected to three freeze-thawing cycles, to remove water-insoluble polymers [17]. Finally, the extracts were freeze-dried, and the yield of the extracts was measured gravimetrically. The fractions produced from birch heart rot and Finnish and Chinese sterile conk, and cultivated mycelium were labeled Heart Rot, F-Chaga, C-Chaga, and IPSsb0–2.5, respectively. Hot water and alkali extracts were marked as HW and 2%, respectively.

### 2.3. Analytical Methods

#### 2.3.1. Total Sugar and Phenolic Contents

Total sugar content of the extracts was measured with the phenol sulfuric acid method adapted for microplate [18]. The quantification was carried using a standard curve of glucose. Total phenolic content was measured with the Folin–Ciocalteau method adapted for microplate, expressed as gallic acid equivalent [19]. Total sugar and phenolic contents were reported as relative weight (%).

#### 2.3.2. Monomer Composition Analysis

The monomer composition of the polysaccharide fractions was measured with GC-FID after hydrolysis with TFA (2 M) and silylation using a protocol described previously [20]. Polysaccharides were hydrolyzed for 6 h at 100 °C. The hydrolysates were filtered, added to internal standard solution (myo-inositol), dried, and silylated. The temperature program was as follows: initial temperature kept at 150 °C for 2 min; increment of 4 °C/min to 210 °C; increment of 40 °C/min to 275 °C; temperature kept at 275 °C for 5 min. The GC-FID apparatus was equipped with a SPB-1 column. The injector and FID temperatures were 210 and 290 °C, respectively. Sugar standard solutions of glucose, mannose, galactose, xylose, rhamnose, arabinose, glucuronic acid, galacturonic acid, glucosamine, and fucose were silylated and analyzed in the same way. The hydrolysates were also analyzed with GC-MS, using the same temperature program and equivalent column. The mass spectrometer operated in EI positive ion mode, with transfer line and ion source temperatures of 280 and 260 °C, respectively, and ionization voltage of 70 eV. The sugars 3-*O*-methyl-galactose and 4-*O*-methyl-glucuronic acid were identified with the diagnostic ion current *m/z* 146.1. They were quantified using the correction factors of galactose and glucuronic acid, respectively.

#### 2.3.3. Molecular Weight Analysis

The molecular weight of polysaccharides was measured with a Waters 2690 HPLC system equipped with a TSK-GMPW column and a refractive index detector. The samples were dissolved in the mobile phase (0.1 M NaNO_3_) to a concentration of 1 mg/mL, and 50 µL of the solutions was injected. The calibration curve was obtained with a series of standard pullulans (Pullulan Kit, Polymer Standards Service, Mainz, Germany). The system flow rate was 0.5 mL/min, and the sample and column temperatures were 40 °C. The molecular weight was reported as molecular weight of the population peak in the chromatogram (M_p_).

#### 2.3.4. Spectroscopical Analysis

Infrared spectra were recorded using a Bruker Vertex 70 spectrometer equipped with a VideoMVP attenuated total reflection accessory. The frequency range 5000–450 cm^−1^ was scanned, with a 2 cm^−1^ resolution.

NMR spectra (^1^H and 2D) were recorded on a Bruker AVANCE III spectrometer, equipped with a Prodigy TCI inverted CryoProbe, operating at 600.20 and 150.92 MHz for ^1^H and ^13^C nuclei, respectively. NMR spectra were recorded at 308 K. A small drop of acetone was added as internal reference (δ^1^H = 2.23 ppm, δ^13^C = 29.6 ppm). The set of experiments was as follows: ^1^H, DQF-COSY, TOCSY (80 ms mixing time), 1D-TOCSY (selective excitation of anomeric proton resonances), HSQC (multiplicity edited), HMBC (one-bond correlation suppression), and NOESY (300 ms mixing time).

## 3. Results and Discussion

### 3.1. Yield, Sugar, and Phenolic Content of Polysaccharides

The polysaccharides of sterile conk obtained from Finnish forest and cultivated mycelium of *I. obliquus* were extracted and characterized. The heart rot of birch infected by this basidiomycete was extracted for assessing the presence of mycelial polysaccharides and for comparison with the polysaccharides extracted from the sterile conk. These three different polysaccharide sources were extracted with hot water and alkali, yielding a hot water extract and an alkali extract for each raw material. The extraction yields and contents of sugars and phenolics of the produced extracts are reported in Table 1. The cultivated strain was obtained from a sterile conk found in a Chinese forest, whose polysaccharides were extracted as well.

The yield of hot water extraction of rot heart wood (Heart Rot HW) was in agreement with the yields obtained after the hot water extraction of birch core [21] and subcritical water extraction of birch sawdust [22]. The sugar contents of Heart Rot HW and alkali extract (Heart Rot 2%) were 19.5% and 44.2% (*w*/*w*), respectively. Overall, the final contents of water and alkali-extractable polysaccharides in the heart rot were 4.4 and 15.4 mg/g, respectively. There are little data available on the polysaccharides extracted from lignocellulosic substrate colonized by the mycelium. Compared to our results, the water extraction of corn biomass colonized by *G. frondosa* mycelium in a bioreactor experiment resulted in lower yield [23]. Conversely, the sugar contents in the heart rot in our study were similar to the amounts obtained after the cultivation of *G. lucidum* mycelium in beech sawdust [24], whereas the levels were lower than those obtained with water extraction from the oak sawdust-based substrate infected with different white-rot fungi [25]. Moreover, a sugar content similar to the level in our study was obtained after the subcritical water extraction of birch sawdust [22]. This is interesting because Koutrotsios and coworkers reported a decrease in the hemicellulose content of lignocellulosic material after white-rot mycelium colonization [26]. *I. obliquus* is able to secrete hemicellulose-degrading enzymes, although, as shown in submerged cultivation conditions, it favors the secretion of ligninolytic enzymes during wood degradation [14]. The yield and sugar content might, therefore, be related to the substrate and fungal species, in addition to the extraction method. However, it should be noted that the dialysis step performed in the present work, responsible for the removal of simple sugars and small oligomers, was not included in the polysaccharide extraction protocols of the aforementioned works.

The hot water extraction of *I. obliquus* sterile conks obtained from the Finnish and Chinese forests showed similar yields (1.1% and 0.9% *w*/*w*, respectively), with only a slight difference, possibly due to the shorter extraction time used for the Chinese sample. The fractions were labeled F-Chaga HW and C-Chaga HW, respectively. On the other hand, the sugar contents of the two extracts were almost identical (27.6% and 26.1% *w*/*w*). The alkali extraction of the Finnish sterile conk (labeled F-Chaga 2%) showed higher yield (9.20% *w*/*w*) compared to the hot water extraction. However, despite the freeze-thawing step, the fraction resulted mainly in substances insoluble in water. Therefore, a second freeze-thawing cycle was performed, and the actual water-soluble fraction was 39.7% (*w*/*w*) of the extract F-Chaga 2%. The insoluble residue produced from the additional freeze-thawing process was not investigated. Both the apparent and actual yields of the extracts are reported in Table 1. Despite the use of alkali, the sugar content of the extract from the alkali extraction was only slightly higher (31.9% *w*/*w*) than the content in the water extract (27.6%).

The cultivated mycelium of *I. obliquus* showed a higher extraction yield, compared to the sterile conk, from extractions with both hot water and alkali media. The sugar contents of the produced fractions were 80% and 76% *w*/*w*% for hot water and alkali extracts, respectively, noticeably higher compared to corresponding extracts from the sterile conk. The decrease in the sugar content of the alkali extract, compared to hot water, was also observed with mycelium cultivated with higher concentrations of sea buckthorn supplement. Moreover, the sugar content of the mycelial hot water extract (IPSsb0–2.5 HW) was in agreement with the hot water extract of mycelium cultivated without supplement [15]. Xu and coworkers reported a comparison of polysaccharides obtained with hot water from cultivated mycelium and sterile conk of *I. obliquus* [10]. Despite the similar protocols used for extraction and purification, the sugar content in the sterile conk fraction was higher (40.5%), while the sugar content of the mycelial extract was lower (64.0%) in their report in comparison with our extracts. On the other hand, our results showed IPS yields obtained with hot water and alkali extraction of 17.2 and 30.8 mg/g starting material, respectively. These values are in agreement with the IPS production of *I. obliquus* [27] and other basidiomycetes [28] reported in the literature. However, it has to be taken into account that in the aforementioned studies, dialysis and precipitation were not used as purification steps after extraction.

The results of Folin–Ciocalteau tests reported in Table 1 showed the presence of phenolic compounds in both hot water and alkali extracts of sterile conk and heart rot, despite the preceding extraction with ethanol and the subsequent dialysis step. No phenolic compound was detected in the polysaccharide fractions obtained from cultivated mycelium. In contrast, phenolics were found in amounts between 4% and 9% *w*/*w* (gallic acid equivalent) in the extracts of heart rot and Chaga sterile conk. The alkali extract of Finnish Chaga (F-Chaga 2%) had the highest level of total phenolic content, almost 19% (*w*/*w*), as determined by the Folin–Ciocalteau test. The compounds contributed to the responses in the Folin–Ciocalteau testwere most likely covalently bound to the extracted polysaccharides. Phenolic deposition and lignification are known mechanisms of plant defense against fungal infection. Both of these phenomena have been observed in birch tissues after infection by *I. obliquus* [29,30]. These molecules are found both in free form and bound to cell wall polymers. Therefore, the phenolic content of the heart rot and Chaga fractions could be attributed to the covalent bond of wood polysaccharides with lignin or phenolic compounds. Previous research has assigned lignin units as the phenolics covalently bound to polysaccharides extracted from the sterile conk of *I. obliquus* [31]. On the other hand, the presence of phenolic compounds in Chaga water extract has been recently explained by the covalent bond between melanin and polysaccharides [32].

### 3.2. Monomer Composition of Polysaccharides

#### 3.2.1. Birch Heart Rot Polysaccharides

The monomer composition of the extracted polysaccharides, expressed as relative molar percentages of individual sugars, is reported in Table 1. Compared to the Chaga extracts (containing 5–15% xylose), the fractions produced from the heart rot of birch infected by *I. obliquus* are clearly distinguished by the predominance of xylose among the monomers (29.8% and 78.0% for hot water and alkali fractions, respectively). Glucose was the second most abundant monomer for both Heart Rot HW and Heart Rot 2% (20.2% and 7.0%, respectively). The xylose relative molar content was slightly higher in Heart Rot HW in comparison with hemicelluloses extracted sequentially from birch sawdust with subcritical water (26.8% and 23.9%, respectively) [22]. The galactose content of Heart Rot HW was also higher (12.2% against 4.1%). The monomer composition of the combined second and subsequent birch sawdust extracts was characterized by the dominance of xylan, which was in general similar to the composition of Heart Rot 2%. However, galactose (3.6%) and particularly glucose (7.0%) contents of Heart Rot 2% were higher than in the birch sawdust extract (2.0% and 1.5%, respectively). The increase in the content of glucose and galactose compared to the amounts in sawdust could be connected to the presence of fungal polysaccharides in Heart Rot HW.

The monomer composition of water extracts of debarked birch biodegraded with *G. lucidum* showed, with the increase in incubation time, an increase in the relative contents of glucose and mannose, while the content of galactose gradually reduced. After the longest colonization time, the glucose and mannose contents of the extract were similar to the levels detected in the Heart Rot HW in our study, while the reported galactose content was higher than what was found in Heart Rot HW [33]. The polysaccharides extracted by Liu and coworkers from the spent solid substrate from the cultivation of *F. velutipes* showed a prevalence of glucose and galactose in their monomer composition, and, compared to our monomer composition results, a higher content of uronic acids. Moreover, their water extract had a higher sugar content (about 88% *w*/*w*). However, the nature of the starting material was not reported [13]. Heart Rot HW had a higher content of 4-O-Me-glucuronic acid (identified with GC-MS, Figure S6), compared to the first birch sawdust extract (3% against 0.7%), while for Heart Rot 2%, it was in agreement with the combined second and following extracts (both about 6%) [22]. The increase in the relative content of uronic acids, compared to birch polysaccharides extracted from uncolonized birch wood, has also already been reported [33].

#### 3.2.2. Sterile Conk Polysaccharides

Compared to the heart rot fractions, the Chaga HW fractions contained xylose at lower proportions (14.6% and 10.9% from Finnish and Chinese Chaga, respectively). Xylose was found in a very low percentage of the total sugars in F-Chaga 2% (5%). Moreover, no 4-O-Me-glucuronic acid was detected in the Chaga fractions (Table 1). Since this monomer is mainly found as a terminal side-chain of xylan, the reduction in the content or even total absence of xylose could be attributed to fungal debranching and degradation of hemicelluloses [34]. The most abundant monomer of the Chaga polysaccharides was glucose, whose relative amount increased from about 36.5% in the hot water extracts to 62% in F-Chaga 2%. This increase is in agreement with the monomer composition of alkali extract of Chaga previously reported [35]. The monomer composition of the hot water extracts from Finnish and Chinese Chaga (F-Chaga HW and C-Chaga HW, respectively) was in agreement with that of polysaccharide fraction from a water extract of Chinese Chaga sterile conk reported by Xu and coworkers [10], although no fractionation was performed in the present work. The C-Chaga HW monomer composition differed from the report mainly in the arabinose content (12.5% against 5.2%). The water extracts from the Finnish and the Chinese Chaga differed in the content of multiple monomers, most noticeably mannose (11.8% against 5.6%, respectively), while the percentages of galactose were similar (15.8% and 16.9%, respectively). 

#### 3.2.3. Mycelium Polysaccharides

Glucose, mannose, and galactose were the main monomers of the polysaccharides extracted from the mycelium of *I. obliquus*, with the marked predominance of the first (62.9% and 55.1% of total sugars for hot water and alkali extracts, respectively) (Table 1). Compared to the results of the present study, similar monomer compositions were reported of the polysaccharide extracts of *I. obliquus* mycelium cultivated in growth media with sea buckthorn press cake (5 and 10 g/L dosages) and without [15]. A lower abundance of glucose and higher proportion of mannose and galactose were reported by Xu and coworkers from the different fractions fractionated from cultivated mycelium [10]. Dominance of glucose over other sugar monomers was also previously observed in polysaccharides extracted from the cultivated mycelia of *I. obliquus* [36,37] and other basidiomycetes [38].

#### 3.2.4. Comparison of the Different Fractions

Among the different fractions analyzed, the highest relative amount of glucose was found in the mycelium extracts (Table 1). In relation to these, similar amounts were found only in F-Chaga 2%, while glucose amounts resulted about 20% less in the sterile conk hot water extracts (62.9% in IPSsb0–2.5 HW against 34.7% in F-Chaga HW). The glucose/xylose ratio (mol/mol) was higher in C-Chaga HW, compared to the ratio in F-Chaga HW (3.5 against 2.4). In the heart rot fractions, xylose was the most dominating sugar monomer, and glucose the second with a relative content lower than that in the sterile conk extracts (18.2% in Heart Rot HW against 34.7% in F-Chaga HW). The glucose/xylose ratio of F-Chaga 2% was lower than IPSsb0–2.5 HW and IPSsb0–2.5 2% (12.2 mol/mol against 43.6 and 32.7, respectively), indicating that, while the alkali extraction of sterile conk showed a relative glucose content similar to mycelium, xylans were still present in the extract.

All the produced fractions showed, with varying abundance, the presence of 3-O-Me-galactose. In our study, the presence of this monomer was first highlighted by NMR spectroscopy (Section 3.5) and then identified in the polysaccharide hydrolysates with GC-MS (diagnostic ion *m/z* 146.1, Figure S5). The occurrence of 3-O-Me-galactose has been reported already in mushroom galactans, particularly of the genus *Pleurotus* [39]. As shown in Table 1, the molar ratio of galactose/methyl-galactose is much higher in Finnish Chaga fractions (14:1 and 15:1 for hot water and alkali fractions, respectively) compared to the values in both heart rot (4:1 and 8:1) and mycelium (10:1 and 8:1). In contrast, the corresponding ratio was 4:1 in hot water extract of Chinese Chaga (C-Chaga HW), highlighting the compositional difference between the two sources of wild Chaga. Our findings differed from the results of Wold and coworkers, who reported a much higher methylation (galactose/methyl-galactose ratio of about 2:1) of the galactans extracted from Chaga [35]. The ratios between galactose and methylated galactose in the galactans extracted from the mushrooms of *P. pulmonarius* and *P. eryngii* were 3:1 and 4:1, respectively [39,40]. Fucogalactan fractions produced from *Agaricus bisporus* var *hortensis* had different galactose/3-O-Me-galactose ratios between 3:1 and 4:1 [41]. The differences in the relative abundance of 3-O-Me-galactose between Finnish and Chinese Chaga and between our results and literature data suggest the species- and strain-specific methylation of galactan, while the difference between C-Chaga HW and IPSsb0–2.5 HW also suggests the influence of the hyphal development stage. The significance of the methylation of galactan for the physiology and development of fungi is still unknown.

### 3.3. Molecular Weight Distribution of Polysaccharides

**Figure 1:**
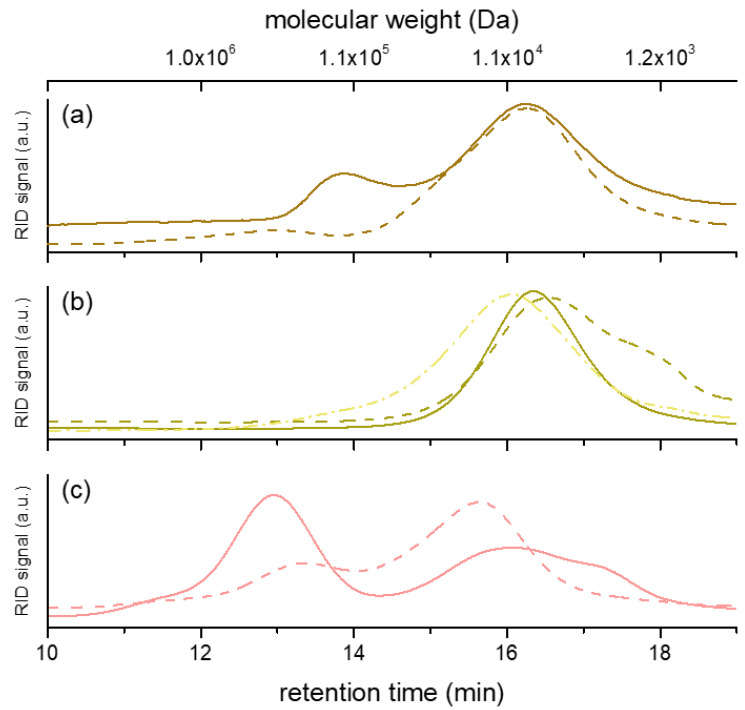
HPSEC chromatograms of the polysaccharide fractions produced from heart rot (**a**), sterile conk (**b**), and cultivated mycelium (**c**). Continuous line represents hot water extracts; dashed line represents alkali extracts. In (**b**), dotted and dashed line represents C-Chaga HW. The correspondence between molecular weight and retention time is based on the calibration curve (Figure S7).

The molecular size and distribution of the polysaccharide fractions were investigated. The HPSEC chromatograms are reported in Figure 1, while the molecular weight values are reported in Table 1. Our results indicated that only polysaccharides in hot water extracts of Chaga (F-Chaga HW and C-Chaga HW) consisted of a single population. Interestingly, the polysaccharides extracted from Finnish and Chinese Chaga differed, although marginally, in molecular size (7.6 and 10.7 kDa, respectively). As could be expected, the use of alkali resulted in polymers of lower M_p_ (6.3 kDa). Our observed values were lower than the M_w_ values reported by Xu and coworkers (32 kDa) and Wold and coworkers (60 kDa) [10,35]. However, this discrepancy could be attributed to the different analytical system used by Xu and Wold and their coworkers, which employed MALLS detection and absolute molecular weight measurement. The polysaccharides extracted with hot water from the heart rot of birch had two distinct populations (1.2 × 10^2^ kDa and 8.3 kDa, respectively), the one of lower molecular weight being more abundant (82% of total peak area). This population was also observed in Heart Rot 2%, where the lower M_p_ population constituted 96% of the polymer area (Table 1). In addition to the monomer composition, the molecular weight of Heart Rot 2% was also in agreement with the values reported for birch xylan [42]. Moreover, the larger polymers of Heart Rot HW had M_p_ in agreement with wood hemicelluloses [43]. Despite the proven ability of white-rot fungi to depolymerize hemicelluloses [33] and the production of xylanase by *I. obliquus* mycelium [14], our results showed similarity in molecular weight between birch heart rot and Chaga polysaccharides. This similarity could be partially attributed to the presence of wood tissue in Chaga, which was also highlighted by the xylose content of the water extracts. However, glucans were more abundant than xylans in the sterile conk fractions; therefore, it could be concluded that Chaga glucans and hemicelluloses had similar molecular weights.

Compared to heart rot and Chaga extracts, the polysaccharides extracted from the cultivated mycelium had a different population profile (Figure 1). In the fraction IPSsb0–2.5 HW, 55% (total peak area) of polymers had a molecular weight of 3.6 × 10^2^ kDa, similar to previous reports on IPS of *I. obliquus* [10]. Despite the presence of sclerotial hyphae in Chaga [1], this population was completely absent from the sterile conk extracts. The other population of IPSsb0–2.5 HW had M_p_ closer to the single population of sterile conk extracts, although slightly higher (Table 1). Noticeably, the population with higher M_p_ of Heart Rot 2% and IPSsb0–2.5 HW had a similar molecular size, although it was present in a very small amount (4% of total area) in the earlier fraction. The use of alkali influenced the molecular weight distribution profile of the extracted mycelial polysaccharides. The chromatogram of IPSsb0–2.5 2% showed a decrease in M_p_ of the population of higher molecular weight, while the population of lower size increased in abundance (from 45% to 74% of total area), possibly due to the alkaline depolymerization of the high M_p_ population. The large size population of the polysaccharides extracted with hot water analyzed in the present study was more abundant and had a higher molecular weight, compared to the corresponding fraction obtained from mycelium cultivated with higher dosages of press cake (5 and 10 g/L) [15]. On the other hand, the HPSEC profile of IPSsb0–2.5 2% was very similar to the profile of the alkali fraction obtained from the same mycelial strain cultivated with higher dosages of sea buckthorn press cake, suggesting a consistent degradation effect [15].

### 3.4. FT-IR Spectroscopy

**Figure 2:**
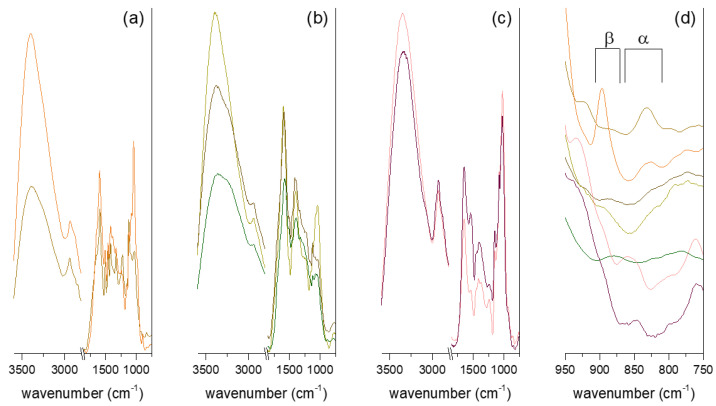
ATR-FT-IR spectra of the polysaccharide fractions. The fractions reported, from top to bottom, are, respectively, (**a**) Heart Rot 2% and Heart Rot HW; (**b**) F-Chaga HW, C-Chaga HW, and F-Chaga 2%; (**c**) IPSsb0–2.5 HW and IPSsb0–2.5 2%. The anomeric region of the stacked spectra is expanded in (**d**). The fractions reported, from top to bottom, are, respectively, Heart Rot HW, Heart Rot 2%, C-Chaga HW, F-Chaga HW, F-Chaga 2%, IPSsb0-2.5 HW, and IPSsb0-2.5 2%.

Infrared spectroscopy is a useful tool for the investigation of chemical groups of extracted compounds, and it has been widely used for the study of plant and mushroom polysaccharides. The ATR-FT-IR spectra of the studied polysaccharide fractions are reported in Figure 2. The stretching of the O-H bond, ascribable to structural hydroxyl groups and absorbed moisture, produced broad bands of 3300–3400 cm^−1^, while the signal at 2930–2940 cm^−1^ was assigned to C-H stretching. The C-O and C-O-C bond vibrations typical of polysaccharide structures were found at around 1040 cm^−1^ for heart rot and Chaga extracts and around 1020 cm^−1^ for the IPS fractions. The same difference in wavenumber can be found when comparing reference hemicelluloses and α-glucan [44]. Noticeably, the C-O and C-O-C vibration group was the most intense of mid-IR region signals only in the mycelial polysaccharides and in the Heart Rot 2% spectra. For the other spectra, the most intense signal in the mid-IR region was the broad band of around 1590 cm^−1^. This signal can be attributed to an overlapping of O-H bending and carboxylate stretching. The presence of aromatic compounds and uronic acids in sterile conk and heart rot extracts (Table 1) could explain the higher intensity of the 1590 cm^−1^ band compared to the polysaccharide structural band. Aromatic skeletal vibrations could contribute to the intensity of this wavenumber band. This was further confirmed by the signal observed around 1120 cm^−1^, which was ascribable to aromatic C-H deformation [45]. The shoulder at 1650 cm^−1^, most prominent in Heart Rot HW but also visible in Heart Rot 2% and partially in F-Chaga 2%, has been assigned to the C=O vibration of lignin bound to hemicelluloses [33]. In addition to the 1590 cm^−1^ band, the FT-IR spectra of heart rot extracts were in good agreement with those reported for hemicelluloses [44].

The FT-IR spectra of Chaga fractions shared some similarities with the Chaga melanin spectrum reported by Wold and coworkers, who suggested 1,8-dihydroxynaphtalene as a monomer unit [32]. In both spectra, the bands around 1590 cm^−1^, 1510 (C=C), 1450 (C-H), 1400 (CO-H), and 1320 cm^−1^ (C-C, C-O aliphatic and aromatic) were observed. Noticeably, the signal around 1400 cm^−1^, which could be due to the presence of polysaccharides, was much less intense in the Chaga melanin spectrum. The bands between 1280 and 1230 cm^−1^ observed in Chaga fractions (Figure 2) were also absent from the Chaga melanin spectrum [32]. The FT-IR spectra of synthetic 1,8-dihydroxynaphtalene (DHN) polymers showed 1611, 1402, and 1284 cm^−1^ bands [46], thus with some agreement with the reported results. However, bands around 1510, 1450, and 1320 cm^−1^ were also observed in lignin-polysaccharide complexes [45], making it, therefore, difficult to unambiguously assign the phenolics in our sterile conk extracts to DHN melanin or lignin.

Differently from the other fractions, the spectra produced by mycelial polysaccharides had strong signals at 1646 and 1542 cm^−1^, which were assigned to protein amide vibrations. The signal ascribable to the β-anomeric bond (around 890 cm^−1^) was clearly observed in the spectra of Heart Rot 2%, C-Chaga HW, and F-Chaga 2%, while it constituted a shoulder in the F-Chaga HW spectrum. Signals ascribable to α-glycosidic linkage were observed in the Heart Rot HW spectrum (830 cm^−1^) and in both mycelial polysaccharide spectra (850–860 cm^−1^). A shoulder ascribable to α-mannans was also found in the IPS fractions around 800 cm^−1^ (Figure 2d) [47]. No clear β-linkage signal was visible in these spectra, indicating the prominence of α-polysaccharides in these fractions.

### 3.5. Nuclear Magnetic Resonance Spectroscopy

**Figure 3:**
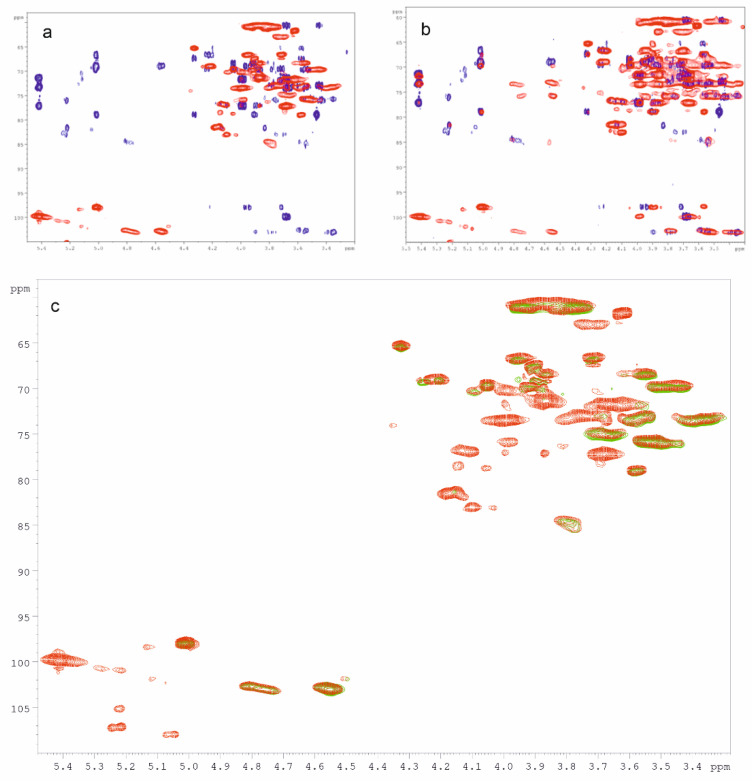
(**a**) HMBC (blue) and HSQC (red) NMR spectra of IPSsb0–2.5 2%; (**b**) HMBC (blue) and HSQC-TOCSY (red) NMR spectra of IPSsb0–2.5 2%; (**c**) HSQC spectra of IPSsb0–2.5 2% (red) and of F-Chaga HW (green). NMR spectra were recorded in D_2_O at 308 K. See Table 2 for signal assignments.

The polysaccharide fractions produced from heart rot of birch, sterile conk, and mycelium of *I. obliquus* were investigated with NMR spectroscopy. The main structural features of the polysaccharides extracted from the mycelium were identified using mono- (^1^H, 1D-TOCSY) and bidimensional experiments (COSY, TOCSY, HSQC, HMBC, NOESY, and HSQC-TOCSY). The ^1^H and HSQC spectra of all the other polysaccharide fractions were then recorded. Due to the high similarity between the HSQC spectra of C-Chaga HW and F-Chaga HW, our structural analysis focused on the latter fraction. The HSQC spectra of IPSsb0–0.5 HW and IPSsb0–0.5 2% were also very similar; therefore, only the latter was considered in the discussion. The HSQC and HMBC spectra of IPSsb0–0.5 2% are reported in Figure 3a. The structural assignments of IPS polysaccharides are reported in Table 2. In the IPS spectra, the strongest anomeric proton signal was found at 5.42 ppm. The HSQC correlation of this proton was found at 99.8 ppm, suggesting an α-configuration. 

The TOCSY spectrum showed ^1^H-^1^H correlations between this anomeric proton and signals at 3.99 and 3.87 ppm. In addition, the spectrum showed a wide correlation around 3.70 ppm, in which different signals were identified with the aid of HSQC and HMBC spectroscopy, and a correlation around 3.45 ppm. In particular, the HMBC correlation 3.68/99.8 ppm allowed the identification of H4 and subsequently of C4 (HSQC correlation 3.68/77.1). The HMBC spectrum showed a correlation 5.42/77.1 ppm cross-confirmed the assignment. Moreover, the HSQC spectrum showed the correlation of protons 3.99 and 3.78 ppm with the broad carbon signal at 60.6 ppm, which was then assigned to C6. Overall, the signal at 5.42 ppm and the identified correlations were in good agreement with glycogen [48]. The predominance of glycogen among the other polysaccharides of IPS fractions could explain the higher intensity of the α-anomeric signal in the FT-IR spectra (Section 3.4). The second most intense anomeric signal of the IPSsb0–0.5 2% ^1^H spectrum resonated at 5.01 ppm. In the HSQC spectrum, it correlated with a carbon signal at 97.9 ppm, suggesting α-configuration. This proton was part of a spin system identified with the combination of TOCSY, 1D-TOCSY, and HSQC spectra. The correlations 4.33/65.3, 4.05/69.5, 3.91/67.3, 3.87/68.4, 3.58/78.9, 3.95/66.5, and 3.72/66.5 were found. This anomeric signal was attributed to the overlapping of the H1 of →6)-α-Gal-(1→6) and →6)-α-3-OMe-Gal-(1→6) units (Table 2). In particular, the correlations 4.33/65.3 and 3.58/78.9 were, respectively, assigned to H4/C4 and H3/C3 of the 3-OMe-Gal unit [40]. In addition, the HSQC spectrum showed the correlation 3.48/56.2, which was assigned to the methoxy group of 3-OMe-Gal and further confirmed by the NOESY correlation 3.48/4.33 ppm (OMe/H4). Two broad proton signals were found in the HSQC spectrum of IPSsb0–2.5 2%, between 4.85 and 4.50 ppm, and they were further investigated. The HMBC correlations 4.81/84.5 (H1/O-substituted C3) and 4.56/69.7 (H1/O-substituted C6) visible in Figure 3a ascribed these broad proton signals to anomers of β-Glc-(1→3) and β-Glc-(1→6) linkages, respectively. The HSQC-TOCSY correlations of these signals are reported in Figure 3b. The two correlations 4.81/84.5 and 4.57/85.0 ppm can be assigned to two different H1/O-substituted C3, respectively, belonging to β-Glc-(1→3) and β-Glc-(1→6) units. While the HSQC-TOCSY (Figure 3b) signal of 4.56/69.7 ppm confirms the →6)-β-Glc-(1→6) unit, the correlation 4.76/69.7 ppm indicated the O-substitution of C6 of the β-Glc-(1→3) unit. The H6 and H6′ signals of the units at 4.56 and 4.76 were assigned to 4.25 and 3.89 ppm with the aid of HMBC (3.89/102.9, H6′/C1), HSQC (H6/C6 and H6′/C6), and 1D-TOCSY (4.56 ppm irradiation). The proton and carbon of position 2 of the β-Glc units were assigned using COSY H1/H2 correlations 4.56/3.35, 4.57/3.55, 4.76/3.40, and 4.81/3.59 ppm, which were cross-confirmed with C1/H2 correlations in the HMBC spectrum. The structural assignments are summarized in Table 2. Terminal β-Glc-(1→3) and β-Glc-(1→6) would be expected to have the H1/C1 HSQC signals overlapping with the →6)-β-Glc-(1→3) and →6)-β-Glc-(1→6), respectively. Noticeably, the HMBC spectrum lacked clear signals ascribable to the →3,6)-β-Glc-(1→ branching point unit. The H1/O-substituted C3 correlation was observed only in the HSQC-TOCSY. However, only the →6)-β-Glc-(1→6) branching point can be clearly excluded. If the β-glucan contains →3,6)-β-Glc-(1→3) branching point units, they would be in little amount. Therefore, it could be inferred from the reported assignments that the β-glucan produced by *I. obliquus* is a mainly linear chain of β-Glc-(1→3) and β-Glc-(1→6) units. Our results are hence in agreement with the results obtained by Wold and coworkers on the polysaccharides extracted from Chaga [35]. The integration of the HSQC anomeric signals suggests a ratio between β-Glc-(1→3) and β-Glc-(1→6) units of 0.8:1. The ratio between glycogen anomeric signal and total β-Glc anomeric signals was 1.7:1.

**Figure 4:**
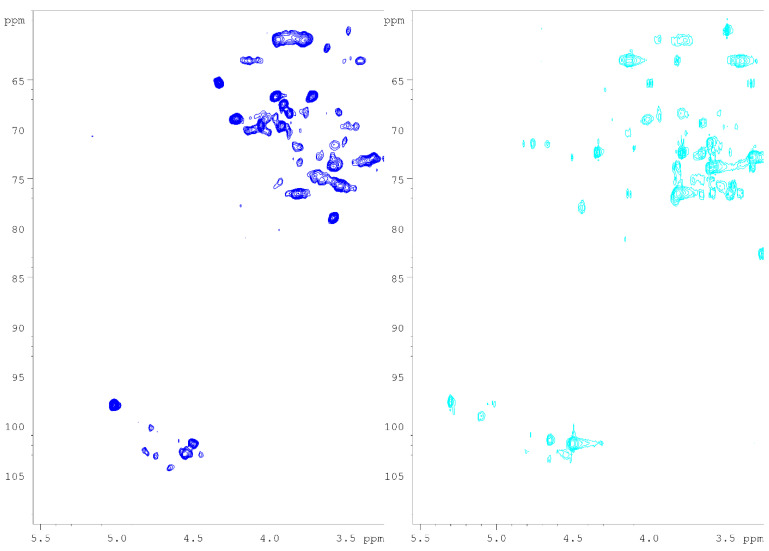
HSQC spectra of Heart Rot HW (blue) and Heart Rot 2% (light blue). NMR spectra were recorded in D_2_O at 308 K. See Table 3 for signal assignments.

The HSQC spectra of Heart Rot HW and Heart Rot 2% are reported in Figure 4. The structural assignments of Heart Rot HW and Heart Rot 2% polysaccharides are summarized in Table 3. 

In the Heart Rot HW spectrum, the H/C correlation ascribable to the anomeric proton of xylan units was found at 4.50/101.7, which was assigned to 4)-β-Xyl-(1→4) unit [49]. The assignment was also confirmed with a xylan standard. The HSQC spectrum of Heart Rot HW showed partially overlapping HQSC correlations around 4.50–4.60/103 ppm. Of these, the correlation 4.53/102.6 was assigned to →4)-β-Glc-(1→4) with the aid of the literature [50]. This glucose unit constitutes the backbone of the hemicellulose glucomannan, which has been extracted previously from birch [51]. Further confirmation of the presence of this polysaccharide in the fraction was provided by the anomeric signal of 4.66/104.1, which was assigned to →4)-β-Man-(1→4) [51]. The presence of acetylated →4)-β-Man-(1→4) units was confirmed by the weak HSQC signals of 5.56/72.6 (O-acetylated C2) and 5.02/75.9 ppm (O-acetylated C3). The corresponding H1/C1 correlations were assigned with the aid of the literature [51] and confirmed with a galactoglucomannan reference compound. The presence of acetyl group was supported by the HSQC correlation 2.19/20.6 ppm. Differently from the hot water extract, the main anomeric signals found in the HSQC spectrum of Heart Rot 2% were ascribable to xylan. The HMBC spectrum of Heart Rot 2% showed the H1/C4 correlation 4.50/76.5, while the HSQC spectrum showed the H4/C4 correlation 3.81/76.5, also confirming the 4.50/101.7 correlation assignment. The correlations 4.65/101.4 and 5.30/97.6 ppm were assigned to →4)-2-OR-β-Xyl-(1→4) and 4-O-Me-α-GlcA-(1→ units, respectively. The substitution in C2 of the xylan unit was mainly ascribable to the methylated glucuronic acid moiety. However, the presence of acetylated 4)-β-Xyl-(1→4) units was hinted by the HSQC signals 4.65/103.3 and 4.61/79.2 ppm, which were tentatively assigned to H1/C1 and H3/C3 of the →4)-3-O-Ac-β-Xyl-(1→4) unit [49,52]. The low intensity of acetylated unit signals could be due to the hydrolytic effect of the alkali extraction medium. The anomeric signal of 5.11/99.0 ppm was assigned to the α-reducing end of xylan [52]. The assignment of this unit to xyloglucan was excluded due to the absence of α-Xyl-(1→6) H1/C6 correlation in the Heart Rot 2% HMBC spectrum. Moreover, the 5.11/99.0 ppm correlation was absent from Heart Rot HW HSQC spectrum. 

Our results showed a complete overlapping in the HSQC spectra of the β-glucan signals of IPS and the anomeric signals of the F-Chaga fractions (Figure 3c). As mentioned above, the HSQC signal of 5.02/97.9 ppm was also present in F-Chaga HW and C-Chaga HW. The preponderance of β-glucan signals in F-Chaga 2% was explained by the preponderance of glucose in the fraction (63% relative mol%). The HSQC spectra of Chaga fractions showed the 4)-β-Xyl-(1→4) unit anomeric signal of 4.50/101.7 ppm. Despite the presence of hyphae in the sterile conk, no glycogen signal was observed in the Chaga extracts. This could be attributed to the metabolic changes undergone by the mycelium during sclerotium formation [53]. The ratio between β-Glc-(1→3) and β-Glc-(1→6) units in Chaga extracts was 0.6:1, irrespective of starting material and extraction method, which was almost the same as the ratio reported by Wold (0.5:1) [35].

**Figure 5:**
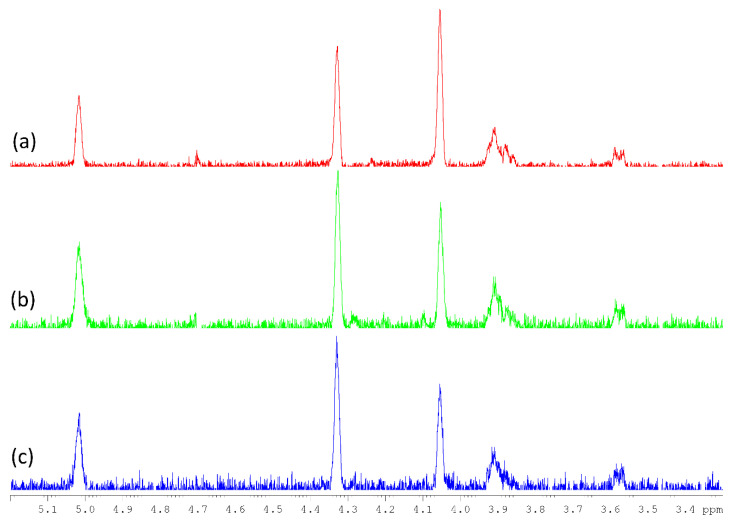
1D-TOCSY spectra of IPSsb0–2.5 HW (**a**, red), F-Chaga HW (**b**, green), and Heart Rot HW (**c**, blue). NMR spectra were recorded in D_2_O at 308 K, with an irradiation frequency of 5.02 ppm. See Table 2 for signal assignments.

As can be noticed in Figure 4, the strong anomeric signal assigned to the 3-O-methylated →6)-α-Gal-(1→6) unit was also remarkably found in Heart Rot HW, while it constituted a weak signal in the Heart Rot 2% HSQC spectrum. The identity between the 5.02/97.9 signals was confirmed by 1D-TOCSY (irradiation frequency of 5.02 ppm). A comparison of the 1D-TOCSY spectra is reported in Figure 5. 

The presence of 3-O-Me-Gal in the fraction hydrolysates was highlighted by GC-MS and quantified with GC-FID. Moreover, the superimposition of the collected HSQC spectra also showed the presence of the mycelial β-glucan signals in the birch heart rot fractions, particularly in Heart Rot HW (Figure S1). The superimposition of the anomeric regions of Heart Rot HW, IPSsb0–2.5 2%, and standard galactoglucomannan is reported in Figure S2. A comparison of the 1D-TOCSY spectra (4.55 ppm irradiation frequency) of Heart Rot HW and F-Chaga HW is reported in Figure S3. The intensity of the O-substituted H3/C3 of the mycelial β-glucan (3.53/75.8 ppm) in the HSQC spectrum of Heart Rot HW was very weak and, therefore, it is absent from Figure 4. Indicatively, based on the anomeric signals, the HSQC spectra showed a ratio between glucomannan and mycelial β-glucan of 1.2:1 in Heart Rot HW, while in Heart Rot 2%, the ratio between xylan and β-glucan was 7.3:1. Moreover, in the Heart Rot HW spectrum, galactose and glucose signals had a ratio close to 1, which decreased to 0.2:1 for Heart Rot 2%. The observed trends were in partial agreement with the results of monomer quantification. The ratio between the mycelial glucan units was 0.7:1 ((1→3):(1→6)) in Heart Rot HW, thus higher than Chaga fractions but lower than IPSsb0–2.5 fractions. The signal of the β-Glc-(1→3) had insufficient intensity in Heart Rot 2% to measure a reliable ratio.

The intensities of the HSQC signals in the aromatic region were too low for any attempt of identification, except for the F-Chaga HW spectrum. Here, some signals ascribable to phenolic compounds were observed, and they are reported in Figure S4. The broad signal of 3.76/56.0 ppm was ascribable to the methoxyl group of aromatic rings. Overlapping with this, the signal of 3.93/56.3 ppm could be assigned to the methoxyl group of condensed aromatic units. It could be concluded that the phenolics present in Chaga extracts had a high degree of methoxylation. The assignments of the HSQC correlations were only tentative (Table S1), and could not unambiguously determine the nature of the phenolic compounds covalently bound to Chaga. However, the results indicated a high degree of substitution with the methoxyl group of the aromatic ring and suggested the presence of hydroxypropyl units, which could be ascribed to lignin or lignans. This evidence was in agreement with the NMR results of Wold and coworkers [35], who also reported a mixture of organic acids and phenolic and methoxyl-substituted compounds as a degradation product of the Chaga melanin or products of lignin degradation [54]. To the best of our knowledge, the structures of the phenolic compounds bound to Chaga polysaccharides remain unclear. Further purification and detailed structural analysis are needed to further clarify their nature.

## 4. Conclusions

Our results showed that the same mixed-linkage (1,3),(1,6)-β-glucan was extracted from both the cultivated mycelium and wild sterile conk of *I. obliquus* (Chaga). The sterile conk of different geographical origins produced polysaccharide fractions of similar composition. These fractions were rich in covalently bound phenolic compounds. On the other hand, the polysaccharide fractions extracted from the cultivated mycelium had almost double the amount of glycogen, compared to β-glucan. In addition, sterile conk polysaccharides constituted a single population of low molecular weight, while the mycelial polysaccharides were clearly polydispersed. The differences in the composition, structure, and molecular weight of mycelial and sterile conk polysaccharides would have a large impact on the biological activities of these fractions. Phenolics covalently bound to polysaccharides could have, for example, a major role in the antioxidant activity of the extracts. Methylated galactan was found in both mycelium and Chaga hot water extracts. Polysaccharides extracted from the heart rot caused by *I. obliquus* were constituted mainly by low molecular weight xylans. However, methylated galactan and (1,3),(1,6)-β-glucan were identified for the first time in the extracts. This evidence suggests that the birch wood used for the production of Chaga could be reutilized for the production of bioactive polysaccharide extracts.

## Figures and Tables

**Table 1:** Extraction yield, sugar, and phenolic contents; monomer composition and molecular weight (M_p_) of the polysaccharide fractions produced from heart rot, sterile conk, and cultivated mycelium.

Fraction	Extraction Yield	Sugar Content	Phenolic Content	Xyl	Gal	Rha	Glc	Man	Ara	Fuc	GalA	GlcA	3-O-Me-Gal	4-O-Me-GlcA	Gal/3-O-Me-Gal	Molecular Weight (M_p_)		
	*w/w* %	*w*/*w* %	*w/w* %	*relative molar* %											*mol/mol*	Population 1 (kDa)	Population 2 (kDa)	Area ^†^ %
Heart Rot HW	2.20	19.49 ± 1.05	9.34 ± 1.14	26.76 ± 0.21	12.15 ± 0.26	3.87 ± 0.05	18.18 ± 0.13	11.84 ± 0.69	7.78 ± 0.13	4.16 ± 0.35	5.39 ± 0.56	0.17 ± 0.07	3.42 ± 0.04	2.96 ± 0.30	4 ± 0	1.2 × 10^2^	8.3	18:82
Heart Rot 2%	3.51	44.23 ± 5.00	6.28 ± 1.67	77.45 ± 1.57	3.63 ± 0.07	n.d. *	7.01 ± 0.06	1.88 ± 0.05	4.50 ± 0.10	n.d. *	2.02 ± 0.02	n.d. *	0.47 ± 0.03	6.28 ± 0.10	8 ± 1	3.6 × 10^2^	8.3	4:96
F-Chaga HW	1.09	27.64 ± 0.19	4.04 ± 0.74	14.58 ± 0.18	15.76 ± 0.2	3.60 ± 0.11	34.68 ± 0.04	11.84 ± 0.19	8.55 ± 0.01	2.64 ± 0.25	6.75 ± 0.27	0.50 ± 0.01	1.11 ± 0.10	n.d. *	14 ± 1		7.6	100
F-Chaga 2%	3.66 (9.20)	31.90 ± 2.70	18.76 ± 2.51	5.11 ± 0.15	5.60 ± 0.11	1.20 ± 0.18	62.27 ± 1.42	11.72 ± 0.17	7.26 ± 0.18	1.5 ± 0.16	4.43 ± 1.98	0.52 ± 0.13	0.40 ± 0.10	n.d. *	15 ± 3		6.3 ^#^	100 ^#^
C-Chaga HW	0.85	26.11 ± 1.73	5.34 ± 0.67	10.91 ± 0.69	16.88 ± 0.24	2.33 ± 0.10	38.33 ± 0.85	5.61 ± 0.07	12.51 ± 0.20	5.49 ± 0.25	3.44 ± 0.25	0.29 ± 0.08	4.2 ± 0.23	n.d. *	4 ± 0		10.7	100
IPSsb0–2.5 HW	2.15	80.02 ± 2.19	n.d. *	1.44 ± 0.02	12.78 ± 0.40	0.17 ± 0.00	62.9 ± 0.46	20.2 ± 0.18	0.45 ± 0.05	1.16 ± 0.08	0.44 ± 0.02	n.d. *	1.3 ± 0.01	n.d. *	10 ± 0	3.6 × 10^2^	10.1 ^#^	55:45 ^#^
IPSsb0–2.5 2%	4.05	76.04 ± 1.38	n.d. *	1.68 ± 0.03	15.47 ± 0.21	0.22 ± 0.01	55.09 ± 0.36	23.42 ± 0.16	0.76 ± 0.04	1.27 ± 0.03	0.20 ± 0.02	n.d. *	1.85 ± 0.04	n.d. *	8 ± 0	2.2 × 10^2^	16.9	26:74

* not detected; # two peaks combined; † percentage of total peak area.

**Table 2:** ^13^C and ^1^H chemical shifts (ppm) for the polysaccharides extracted from *I. obliquus* mycelium.

Type of linkage	C1/H1	C2/H2	C3/H3	C4/H4	C5/H5	C6/H6	O-CH_3_
→6)-β-Glc-(1→6)	102.9 4.56	73.1 3.35	75.8 3.53	68.3 3.62	73.2 3.71	69.7 4.25/3.89	
→3)-β-Glc-(1→6)	102.7 4.57	72.9 3.55	85.0 3.77	68.2 3.54	71.3 3.67	60.6 3.94/3.76	
→6)-β-Glc-(1→3)	103.0 4.76	73.6 3.40	75.8 3.53	68.3 3.62	73.2 3.71	69.7 4.25/3.89	
→3)-β-Glc-(1→3)	102.7 4.81	73.2 3.59	84.5 3.81	68.2 3.54	71.3 3.67	60.6 3.94/3.76	
→6)-α-3-OMe-Gal-(1→6)	97.9 5.02	67.3 3.91	78.9 3.58	65.3 4.33	68.4 3.87	66.5 3.95/3.72	56.2 3.48
→6)-α-Gal-(1→6)	97.9 5.02	67.3 3.91	69.5 4.05	n.a. *	68.4 3.87	66.5 3.95/3.72	
→4)-α-Glc-(1→4)	99.8 5.42	69.7 3.45	73.2 3.99	77.1 3.67	71.5 3.87	60.6 3.94/3.76	

* not assigned.

**Table 3:** ^13^C and ^1^H chemical shifts (ppm) for the polysaccharides extracted from birch heart rot caused by *I. obliquus*.

Fraction	Type of linkage *	C1/H1	C2/H2	C3/H3	C4/H4	C5/H5	C6/H6	O-CH_3_
HW (2%)	→6)-α-3-OMe-Gal-(1→6)	97.9 5.02	67.3 3.91	78.9 3.58	65.3 4.33	68.4 3.87	66.5 3.95/3.72	56.2 3.48
HW	→4)-β-Glc-(1→4)	102.6 4.53	72.8 3.23	n.a. ^$^	76.4 3.82	n.a. ^$^	60.6 3.94/3.76	
HW	→4)-β-Man-(1→4)	104.1 4.66	70.1 4.13	n.a. ^$^	76.4 3.82	n.a. ^$^	60.6 3.94/3.76	
HW	→4)-3-OR-β-Man-(1→4)	100.1 4.79	69.1 4.22	75.9 5.02	n.a. ^$^	n.a. ^$^	60.6 3.94/3.76	
HW	→4)-2-OR-β-Man-(1→4)	99.7 4.87	72.6 5.56	n.a. ^$^	n.a. ^$^	n.a. ^$^	60.6 3.94/3.76	
HW, 2%	→4)-β-Xyl-(1→4)	101.7 4.50	72.8 3.33	73.7 3.59	76.5 3.81	62.8 4.13/3.40		
2%	→4)-2-OR-β-Xyl-(1→4)	101.4 4.65	72.8 4.50	n.a. ^$^	76.1 3.83	62.8 4.13/3.40		
2%	→4)-3-OR-β-Xyl-(1→4) ^#^	103.3 4.65	n.a. ^$^	79.2 4.61	n.a. ^$^	62.8 4.13/3.40		
2%	→4)-α-Xyl	99.0 5.11	n.a. ^$^	n.a. ^$^	76.5 3.81	62.8 4.13/3.40		
2%	4-O-Me-α-GlcA-(1→	97.6 5.30	71.5 3.60	72.3 3.79	82.5 3.26	72.3 4.35		56.1 3.47

* R indicates acetyl group (OAc 2.19/20.6 ppm) or, in the case of 2-OR-β-Xyl, 2-O-linked 4-O-Me-α-GlcA; ^#^ tentative assignment; ^$^ not assigned.

## Data Availability

Data sharing not applicable.

## Supplementary Materials

The following are available online at https://www.mdpi.com/2309-608X/7/3/189/s1, Figure S1: HSQC spectra of F-Chaga HW, F-Chaga 2%, Heart Rot HW, and Heart Rot 2%. Figure S2: Anomeric region of the HSQC spectra of IPSsb0–2.5 HW, Heart Rot HW, and galactoglucomannan standard. Figure S3: 1D-TOCSY spectra of F-Chaga HW and Heart Rot HW. Figure S4: HSQC spectrum of F-Chaga HW, with zoom on the aromatic region. Figure S5: GC-MS fragmentation pattern of persilylated 3-O-Me-Galactose. Figure S6: GC-MS fragmentation pattern of persilylated 4-O-Me-Glucuronic acid. Figure S7: HPSEC calibration curve with pullulan standard set. Table S1: Tentative assignments of the lignin-correlated signals found in the HSQC spectrum of F-Chaga HW.
