# Supplementary material for: Comparison of Polysaccharides Extracted from Cultivated Mycelium of Inonotus obliquus with Polysaccharide Fractions Obtained from Sterile Conk (Chaga) and Birch Heart Rot
Source: J Fungi (Basel). 2021 Mar 8;7(3):189. doi: 10.3390/jof7030189 (PMC8000984; doi:10.3390/jof7030189)
Supplement: Supplementary file 1 [file jof-07-00189-s001.pdf]

Comparison of polysaccharides extracted from cultivated mycelium of *Inonotus obliquus* with polysaccharide fractions obtained from sterile conk (Chaga) and birch heart rot

Gabriele Beltrame[1], Jani Trygg[1], Jarl Hemming[2], Zenghua Han[3], Baoru Yang[1,*]

[1] Food Chemistry and Food Development, Department of Life Technologies, University of Turku, Itäinen Pitkäkatu 4, FI-20520 Turku, Finland
[2] Wood and Paper Chemistry, Åbo Akademi University, Porthaninkatu 3, FI-20500 Turku, Finland
[3] Institute of Microbiology, Heilongjiang Academy of Sciences, Zhaolin Street 68, CN-150020 Harbin, China
* Correspondence: B. Yang: baoru.yang@utu.fi. Postal address: Itäinen Pitkäkatu 4 C, 20520 Turku, Finland.

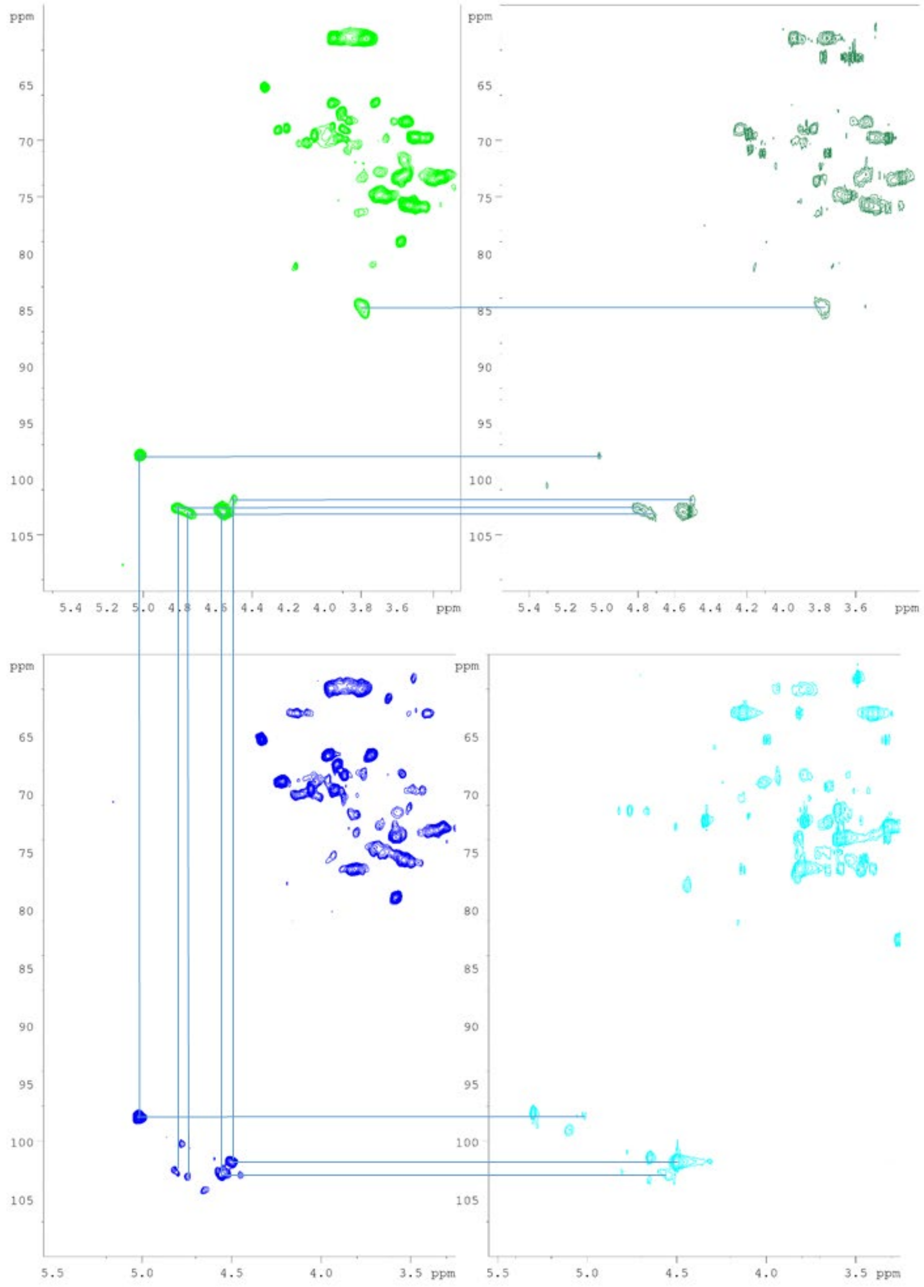

Figure S1. HSQC spectra of F-Chaga HW (green), F-Chaga 2% (dark green), Heart Rot HW (blue), and Heart Rot 2% (light blue). NMR spectra were recorded in D_2O at 308 K. See **Table 2** and **Table 3** for signal assignments.

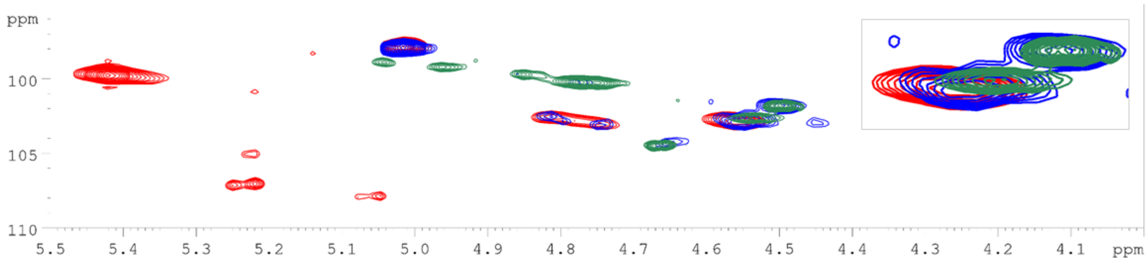

Figure S2. Anomeric region of the HSQC spectra of IPSsb0-2.5 HW (red), Heart Rot HW (blue), and galactoglucomannan standard (olive). NMR spectra were recorded in D_2O at 308 K. See **Table 2** and **Table 3** for signal assignments.

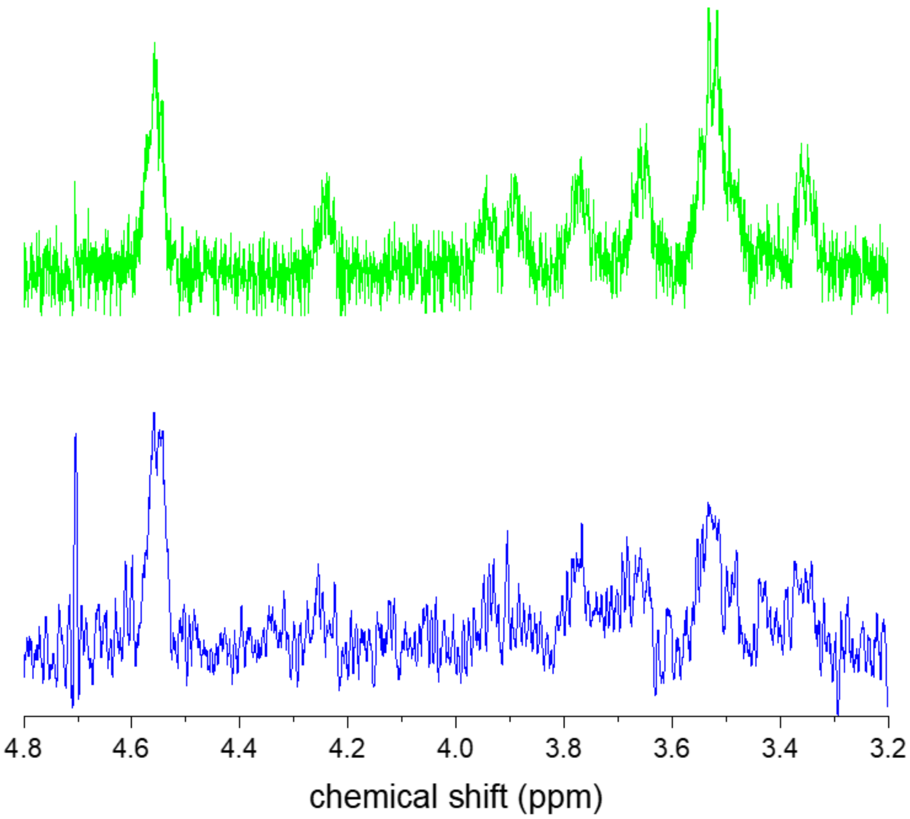

Figure S3. 1D-TOCSY spectra of F-Chaga HW (green), and Heart Rot HW (blue). NMR spectra were recorded in D_2O at 308 K, with an irradiation frequency of 4.55 ppm. See **Table 2** for signal assignments.

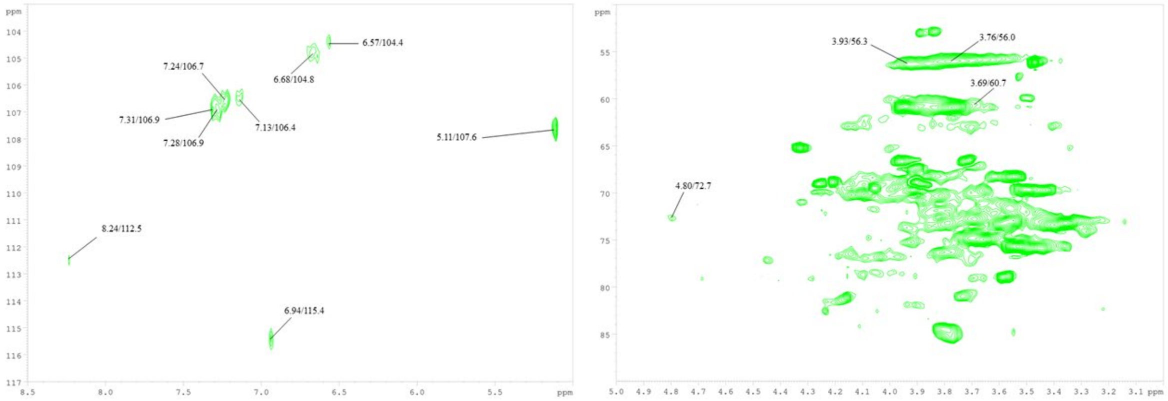

Figure S4. HSQC spectrum of F-Chaga HW, with zoom on the aromatic region (left). NMR spectrum were recorded in D_2O at 308 K.

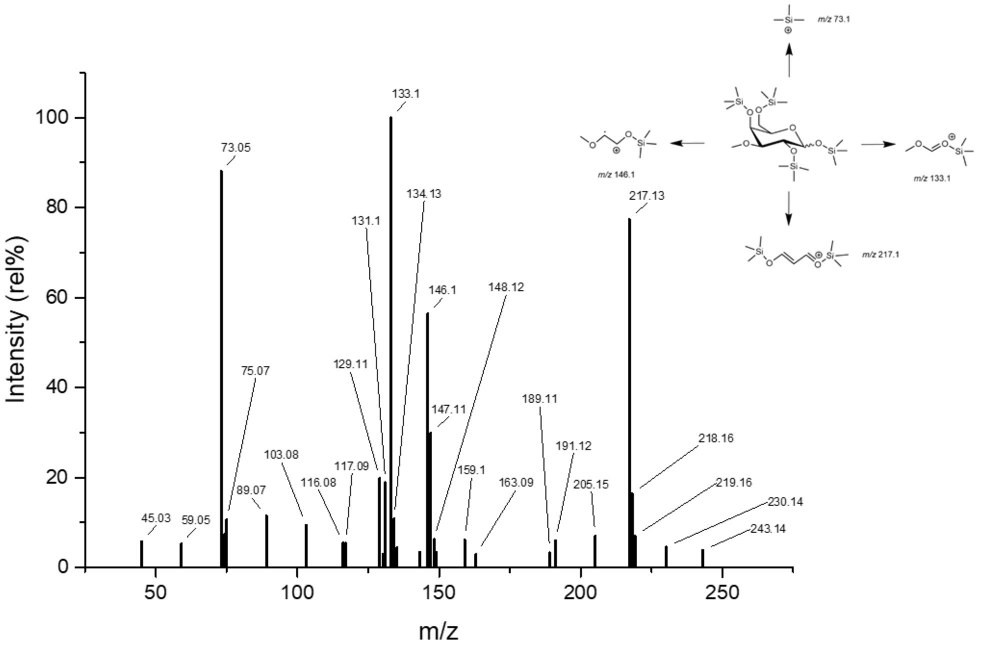

Figure S5. GC-MS fragmentation pattern of persilylated 3-O-Me-Galactose.

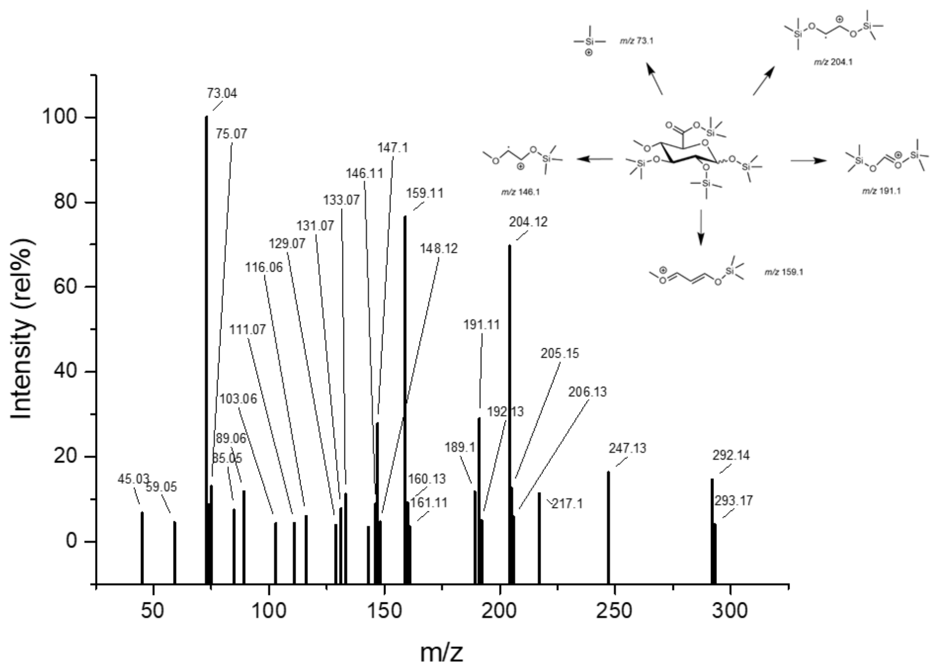

Figure S6. GC-MS fragmentation pattern of persilylated 4-O-Me-Glucuronic acid.

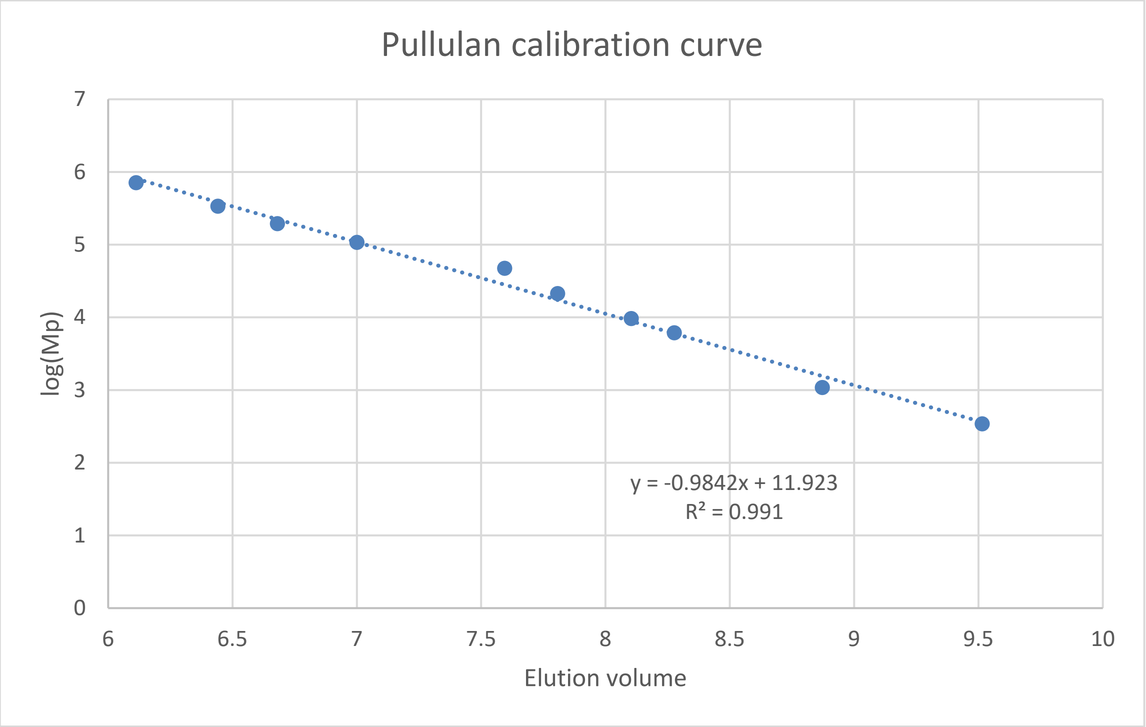

Figure S7. HPSEC calibration curve with pullulan standard set.

Table 1. Tentative assignments of the lignin-correlated signals found in the HSQC spectrum of F-Chaga HW.

^{1}H	^{13}C	Assignment
3.76	56.0	OMe
3.93	56.3	OMe condensed aromatic units
3.69	60.7	Hγ/Cγ of β-O-4 substituted units
4.8	72.7	Hα/Cα of β-O-4 substituted units
6.57	104.4	H2/C2 of free 4-OH syringyl units
6.68	104.8	H2/C2 of syringyl units
7.31	106.9	H2/C2 of α oxidized syringyl units
5.11	107.6	H/C aryl enol ether
7.67	113.7	H2/C2 of α oxidized guaiacyl units
6.94	115.4	H2/C2 of guaiacyl units
